# Outcome after Surgery of Lumbar Spinal Stenosis: A Randomized Comparison of Bilateral Laminotomy, Trumpet Laminectomy, and Conventional Laminectomy

**DOI:** 10.3389/fsurg.2016.00019

**Published:** 2016-04-08

**Authors:** Kaveh Haddadi, Hamid Reza Ganjeh Qazvini

**Affiliations:** ^1^Department of Neurosurgery, Diabetes Research Center, Emam Hospital, Mazandaran University of Medical Sciences, Sari, Iran; ^2^Department of Neurosurgery, Faculty of Medicine, Mazandaran University of Medical Sciences, Sari, Iran

**Keywords:** lumbar stenosis, outcome, laminectomy, laminotomy, trumpet

## Abstract

**Background:**

Laminectomy is the traditional operating method for the decompression of spinal canal stenosis. New partial decompression processes have been suggested in the treatment of lumbar stenosis. The benefit of a micro surgical approach is the chance of an extensive bilateral decompression of the spinal canal or foramen at one or numerous levels, through a minimal para-spinal muscular separation.

**Purpose:**

To match the safety and the clinical consequences after a bilateral laminotomy, laminectomy and trumpet laminectomy in patients with lumbar spinal stenosis who were randomized to one of three treatment groups.

**Study design:**

Prospective study.

**Methods:**

One hundred twenty consecutive patients with 227 levels of lumbar stenosis without significant herniated discs or instability were randomized to three treatment groups [bilateral laminotomy (Group 1), laminectomy (Group 2), and trumpet laminectomy (Group 3)]. Perioperative parameters and complications were documented. Symptoms and scores, such as a visual analog scale (VAS), Oswestry Disability Index, and patient satisfaction, were assessed preoperatively at 3, 6, and 12 months after surgery. Adequate decompression was achieved in all patients on the basis of surgeon satisfaction.

**Results:**

The global complication rate was lowest in patients who had undertaken bilateral laminotomy (Group 1). The minimum follow-up of 12 months was achieved in 100% of patients. Matched with that experience in Group 1, but, with more remaining back and leg pain was found in Group 2, 3.85 ± 0.28 and 1.60 ± 0.44, respectively and 3.24 ± 0.22 and 2.44 ± 0.26 in Group 3, respectively compared with 1.84 ± 0.28 and 1.25 ± 0.12 (Group 1) at the 1-year follow-up assessment (*p* < 0.05). It was the same for the ODI scores, which reached 14 ± 8% (Group 1), 28 ± 12% (Group 2), and 26 ± 16 after 12 months of surgery (Group 3) (significant, *p* < 0.01 compared with preoperative scores). Patient satisfaction was higher in Group 1, with 7.5, 20, and 25% of patients displeased (in Groups 1, 2, and 3, respectively; *p* < 0.01).

**Conclusion:**

Bilateral Laminotomy is certified acceptable and harmless in decompression of lumbar stenosis, causing a highly significant decrease of symptoms and disability.

## Introduction

Lumbar spinal stenosis (LSS) is commonly seen in the elderly especially owing to the aging of the spine. Growing in the facet joints, ligamentum flavum hypertrophy, disc degeneration, and osteophytes cause the spinal canal to constrict and accordingly result in spinal cord and nerve root compression (1). Chief symptoms are low back pain and leg pain worsened by walking and numbness in the legs (2). Surgery must be pragmatic on patients who do not respond to conventional treatment (3). Minimally invasive approaches are cumulative in number as the equipment advances. Two of these minimally invasive methods are the bilateral laminectomy and trumpet laminectomy (4). This study provides a comparative analysis of the clinical and radiological results obtained in classic decompressive laminectomy cases using these two approaches (1, 2, 4).

Wide laminectomy is the most common surgical approach for the decompression of spinal canal stenosis. This standard technique permits maximal operative contact for the bilateral neural canal and/or foraminal decompression. There is subsequent extensive damage of the paraspinal muscles, the interspinous ligament, the supraspinous ligament, posterior bone rudiments, and, occasionally, the capsular facet.

Growing information of the pathoanatomy, joined with high-resolution imaging, has permitted a detailed localization of nerve root compression, which generally happens near the intervertebral space and the expanded ligamentum of flavum (5–7). Numerous authors have planned more custom-made and fewer invasive techniques in the handling of acquired lumbar stenosis, including micro hemi laminotomy, interlaminar micro decompression, inter segmental micro decompression, recapturing micro laminoplasty, and segmental micro sub laminoplasty (8–10). In particular, bilateral (11–13) and unilateral laminotomy for bilateral decompression (14–17) have been described.

In Japan, one of the public processes for micro decompression of the lumbar spinal canal is trumpet laminectomy fenestration. This method conserves posterior lumbar associate constructions aimed at spinal constancy and avoids weakening of the paraspinal muscle, allowing for enhanced disclosure of intraspinal nerves and sufficient decompression of the spinal channel. The indications for trumpet laminectomy micro decompression are parallel to those of standard lumbar decompression. Patients with degenerative stenosis and major leg discomfort, who have not responded to conventional methods, are ideal surgical applicants, notwithstanding the amount of sections of lumbar involvement (18).

The benefit of a micro surgical method is the opportunity for an extensive bilateral decompression of the spinal canal or foramen, through a slight paraspinal muscular separation. Therefore, it is possible to stabilize the spine while protecting the vital soft tissues and bones, at the same time resecting the bilateral pathologies compressing on the spinal canal or foramina (14).

The results so far have been hopeful, with success rates as high as 90%. However, the biggest of these clinical series involved few people, not necessarily with the same symptoms, and the results were either retrospective or without a control group. In the few qualified studies, investigators did not find a significant advantage related to a less invasive method compared with laminectomy (19–21) but reported a higher occurrence of preoperative (neurological) morbidity.

As a result, the authors of review articles concluded that laminectomies should be reserved for cases in which the disease was far less severe or for specific subgroups of patients (22–24). Comparative data obtained in a population of a sufficient size, however, have not been reported in a prospective trial (22). The purpose of our prospective study was to compare the safety and the clinical outcomes after bilateral laminotomy, laminectomy, and trumpet laminectomy in patients with LSS who were randomized to one of the three action groups.

## Materials and Methods

We designed the first prospective study to match the safety and consequences of bilateral laminotomy and trumpet laminectomy by laminectomy.

Among 163 patients, 120 patients (mean age 66 ± 8 years, range 46–85 years) by lumbar spinal stenosis unresponsive to tolerable conservative management were selected throughout a 36-month period. The subsequent inclusion criteria were used: (1) indications of neurogenic claudication or radiculopathy; (2) neuroimaging signs of degenerative stenosis; (3) lack of related pathological matters such as disc herniation or instability; and (4) no presence of surgery for lumbar stenosis or fusion. Symptoms were measured as intractable to non-surgical organization if traditional trials, principally non-steroidal anti-inflammatory drugs and somatic therapies, had been used for at least 12 weeks without enough improvement. Unlike preceding studies in which the authors permitted discectomy to be a portion of the decompression (8, 10, 12, 20, 25) or involved fusion procedures (20), we tried to study a uniform population. (1) We left out from the result analysis nine patients who had necessary discectomies due to substantial intraoperative distinguished discogenic nerve compression, which had not been recognized on preoperative imaging and replace them with nine stenotic patients again. (2) We selected patients with back and leg visual analog scale (VAS) above score seven. Spinal instability was demarcated as sagittal-plane translation of 5 mm or more recognized on flexion–extension X-ray (23, 26). Patients exhibiting stable spondylolisthesis or having a past of surgery for herniated lumbar discs were excepted. Finally, diabetic patients and osteoporotic or heavy smoker patients were excluded from the study.

### Preoperative Assessment

All patients undertook a consistent neurological and clinical valuation, and pain was measured for the low back and the legs according to a self-assessment 10-point VAS (27). Disability was assessed using the ODI (Oswestry Disability Index).

Radiological/neuroimaging examination involved MR imaging for all patients and post myelography CT scanning for documentation of the involved segments in some patients with MRI Suspicious results has done. In the majority of patients, we detected multi segmental stenosis, with mandatory decompression of 227 levels overall (mean 1.891 per patient). The L3–L4 and the L4–L5 levels were most commonly involved [in 96 (42.2%) and 95 (41.8%) of cases, respectively].

### Randomization Plan

Every patient’s admittance numeral was used to enable the randomization of individual information. If a person met the inclusion criteria when given to the admitting doctor and informed consent was obtained, an obscured computer randomization tilt was used to allocate the patient to one of the action groups: bilateral laminotomy (Group 1), conventional laminectomy (Group 2), and trumpet laminectomy (Group 3).

### Operating Processes

**Figure 1:**
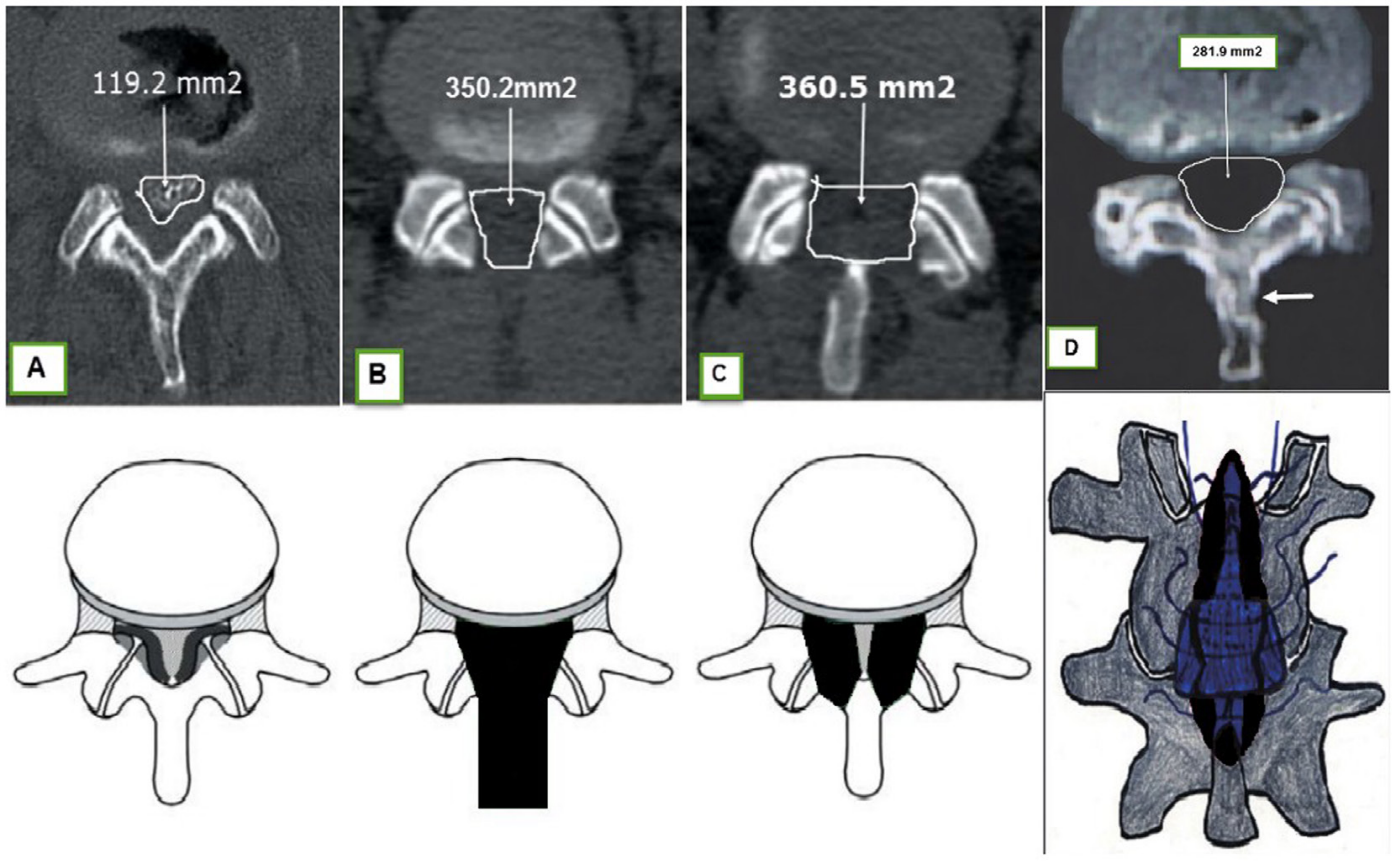
**Upper**: axial postoperative CT scans. Reminder the (posterior) salvation of the facet joints as a result of establishment of an undercutting technique. **(A)** (normal), **(B)** (Group 2), **(C)** (Group 1), and **(D)** (Group 3) arrow showed the replacement part of resected lamina in trumpet laminectomy. Lower: schematic illustrations again demonstrating the surgical corridors associated with each group (dark) [**(A–D)** (28)].

All patients undertook surgery after administration of general anesthesia in the prone position. An operative microscope was used in all cases. The operation was done in a consistent way. The surgical method was represented by axial postoperative CT scans in Figure 1. The three methods used in the groups had usually been achieved at our organization in the 3 years prior to the study.

#### Bilateral Laminotomy (Group 1)

The bone of the lower feature of the cephalad lamina and, to a small extent, from the superior feature of the inferior lamina was resected, and following flavectomy was completed to expose the canal. The medial feature of the facet joint was resected to expand the lateral recess. The spinous process, the supra- and interspinous ligaments, and a considerable percentage of the lamina stayed conserved (Figure 1C) (11–13, 29).

#### Laminectomy (Group 2)

The spinous process and the laminae of the complicated segment(s) as well as the medial features of the facet joints were resected (30). Singular attention was taken in all three groups to diminish facet joint resection by using a denting procedure. Suction drains were regularly located. CT scans after surgery were acquired in all patients before release to evaluate the competence of the decompression (Figure 1B).

#### Trumpet Laminectomy (Group 3)

The spinous process was uncovered 20-mm wide, centered at the interspinous level to be decompressed. While protecting the supraspinous ligament and the interspinous ligament, the paravertebral muscle and the capsular facet were left totally undamaged.

A sharp midline cut was done with a 2-mm high-speed drill, preserving the ligamentous supplement to the rostral part of the spinous process (Figure 1D) (28). At that time, the tip of the spinous process was released by a dissector, and the base of it was cut by a high-speed drill. Similar practice for multiple level decompression is essential. The working field was released from 1/3 to 1/2 of the caudal part of the complicated laminae. The size of the trumpet laminectomy is usually 2 cm. Flavectomy was done for patients with hypertrophic yellow ligament, afterward expanding the neural materials, then the split spinous process was rebuilt and relocated (28).

### Assessment of Surgical Procedure Morbidity

Intraoperative factors like the size of the skin cut, duration of the technique, EBL (estimated blood loss), and intraoperative problems were recognized in a consistent form in the operative area. These records were evaluated relative to the amount of decompressed points. Perioperative morbidity encompassed reoperations in 1 month and the occurrence of an augmented postoperative radicular discrepancy such as neural damage. The procedural difficulty of the technique was evaluated by the surgeon on a 10-point scale.

### Outcome Valuation

Pain (VAS score), ODI scores, and general success rate (0% no success; 100% complete success) were logged at follow-up checkups at 1, 6, and around 12 months after the operation. To assess patient satisfaction with the postoperative outcome, the PSI (a modified sub item of the NASS outcome questionnaire) was used (31).

Patients showing substantial residual or recurrent symptoms undertook postoperative MR imaging and flexion–extension radiography. In cases of instability, residual or adjacent-level stenosis, or lumbar facet syndrome, surgical intervention took place and was documented.

### Statistical Analysis

The unpaired Student’s *t*-test, chi-square test, and Fisher exact test were used as appropriate to analyze differences in the preoperative clinical and demographic characteristics (age, sex ratio, duration of symptoms, clinical presentation, VAS, and ODI) in the intra operative variables and in clinical outcome variables between groups (VAS, ODI, and PSI scores as well as reoperations). The paired Student’s *t*-test and Wilcoxon signed-rank test were used to analyze variations over time inside each group. Statistical significance was established at a likelihood rate of <0.05.

## Results

Forty patients individually were randomized to one of the three groups. There were no significant dissimilarities in the preoperative individuals in the three groups (Table 1). The VAS preoperative overall pain for back and legs were recorded prospectively. The patients suffered from neurogenic claudication for a mean of 24.6 months. The overall ODI disability score was 75.3%. There were no intergroup significant variances in the preoperative pain features (Table 2).

**Table 1:** **Clinical and demographic data achieved in randomized patients with lumbar spinal stenosis^a^**.

Parameters	Group 1	Group 2	Group 3
Number of cases	40	40	40
Mean age (year)	68 ± 9	67 ± 8	68 ± 8
Male/female ratio	18/22	24/16	23/17
Mean BMI (kg/m^2^)	26 ± 4	24 ± 5	26 ± 6
Level (no. of case)			
L1–L2	1	2	1
L2–L3	8	9	7
L3–L4	32	33	31
L4–L5	33	32	30
L5–S1	2	3	3
Symptom (no. of case)			
LBP	34	35	34
Neurogenic claudication	38	40	39
Numbness	32	30	28
Leg pain	33	36	35
Mean duration of symptom			
LBP	66 ± 70	65 ± 68	68 ± 72
Leg pain	22 ± 33	18 ± 25	20 ± 36
Neurogenic claudication	26 ± 32	22 ± 36	26 ± 33

*LBP, low-back pain*.

*^a^Mean values are presented as the means 6 SDs*.

**Table 2:** **Patients pain assessment before and after surgery**.

Parameters	Group 1	Group 2	Group 3
VAS back pain before surgery	8.01 ± 1.36	8.22 ± 1.75	8.42 ± 1.33
VAS back pain 1 month after surgery	3.22 ± 0.33	5.08 ± 1.46	5.85 ± 1.32
VAS back pain 6 months after surgery	1.74 ± 0.35	4.23 ± 0.86	4.33 ± 0.64
VAS back pain 12 months after surgery	1.84 ± 0.28	3.85 ± 0.28	3.24 ± 0.22
VAS leg pain before surgery	7.34 ± 1.22	7.52 ± 1.44	8.11 ± 1.22
VAS leg pain 1 month after surgery	4.08 ± 0.62	3.46 ± 0.38	5.33 ± 0.75
VAS leg pain 6 months after surgery	2.88 ± 0.54	2.33 ± 0.36	3.2 ± 0.48
VAS leg pain 12 months after surgery	1.25 ± 0.12	1.6 ± 0.44	2.44 ± 0.26
ODI before surgery	73 ± 16%	75 ± 33%	78 ± 30%
ODI 1 month after surgery	38 ± 16%	48 ± 26%	44 ± 12%
ODI 6 months after surgery	20 ± 12%	31 ± 14%	30 ± 22%
ODI 12 months after surgery	14 ± 8%	28 ± 12%	26 ± 16%

### Intraoperative Parameters

Spinal decompression was sufficiently attained in all surgical cases. Thus, the planned technique was followed in all patients. The time of operation was significantly long for Group 3. The EBL was the lowest in patients who undertook the bilateral method in Group 1. No patient required a blood transfusion in Group 1.

The skin incision was longer in Group 3 patients compared with those who experienced laminotomy and laminectomy. The procedural difficulty of the techniques was rated maximum in Group 3 (Table 3).

**Table 3:** **Intraoperative parameters restrained during surgical decompression**.

Parameters	Group 1	Group 2	Group 3
Duration of operation (min/level)	58.6 ± 3.6	70.16	73.2 ± 10.6*
EBL ± (ml/level)	125 ± 46 cc	240 ± 65 cc**	220 ± 80 cc**
Length of skin incision (cm/level)	2.5 ± 0.6	3.8 ± 0.8∞	4.2 ± 1.2^∞^
Difficulty of OP (range 0–10)	5.3 ± 1.2	6.2 ± 0.8	7.2 ± 0.9^●^

*p < 0.01 compared with Group 1.

**p < 0.01 compared with Group 1

*^∞^*p* < 0.001 compared with Group 1*.

*^●^p < 0.05 compared with Group 1*.

### Morbidity of Surgery

We did not have perioperative deaths. Of totally treated levels unintentional durotomy happened in Group 1, two levels; Group 2, five levels; and Group 3, eight levels. Dural tears were not obviously related to postoperative morbidity, but they were with increased duration of surgery and augmented EBL. In the worst cases, direct stitching was done using special micro instruments. No subsequent postoperative CSF fistula was detected. An epidural hematoma needing reoperation was recognized on MR imaging in Group 2 and one in Group 3 patients, and four Group 2 patients and two in Group 3 who presented with postoperative urinary retention (six cases totally). Increased radicular pain (one case in each group) or progressive radicular deficit (one case; Group 2) were perceived.

One wound infection was noted in a laminectomy-treated patient after removal of an epidural hematoma requiring a second operation and antibiotic therapy (Table 4) and one in Group 3.

**Table 4:** Summary of perioperative complications*.

Complication	Group%-1	Group%-2	Group%-3
Incidental durotomy	1 (2%)	5 (12.5%)	4 (10%)
Increased radicular pain	1 (2%)	1 (2%)	1 (2%)
Wound infection	0 (0%)	1 (2%)	1 (2%)
Epidural hematomas	0 (0%)	1 (2%)	1
Total (no. of patients)	2 (4)	6 (15)**	6 (15)**

*Complications were calculated per patient, that is, the number of complicated cases per group was evaluated by whether one patient ached from a single or multiple complications.

**p < 0.05 compared with Group 1.

Overall, the perioperative morbidity rate, including the clinically occult incidental durotomies, was less in Group 1 (4%) than in Group 3 (15%; *p* < 0.05 compared with Group 1) or Group 2 (15%) (Table 4).

### Follow-Up

Follow-up records for consequence analysis were made at 1, 6, and 12 months after the operation. In most patients, the last valuation was directed 12–18 months postoperatively (mean follow-up period 14.3 months). In that time, two patients died of unconnected reasons 12 months after surgery, one in Group 1 and one in Group 2. All patients were followed up.

Therefore, 120 patients were followed up over at least a 12-month period. In six patients, the latter part of the questionnaire (PSI) was inadequately completed, preventing analysis.

### Assessment of Pain

Operating decompression leads to an intense decrease of total pain in all three groups (*p* < 0.001). Matched with that experience in Group 1, but, with more remaining back and leg pain was found in Group 2, 3.85 ± 0.28 and 1.60 ± 0.44, respectively, and 3.24 ± 0.22 and 2.44 ± 0.26 in Group 3, respectively, compared with 1.84 ± 0.28 and 1.25 ± 0.12 (Group 1), respectively, at the 1-year follow-up assessment (*p* < 0.05). The most obvious symptom of lumbar stenosis, neurogenic claudication improved in 91% of patients in Group 1 compared to 84 and 82% in Groups 2 and 3 (*p* < 0.05), respectively.

### Assessment of Disability

The same was true for the ODI scores, which reached 14 ± 8% (Group 1), 28 ± 12% (Group 2), and 26 ± 16 after 12 months after surgery (Group 3) (significant, *p* < 0.01 compared with preoperative scores; Table 2).

### Necessary Reoperations

Postoperative CT scanning established adequate decompression in all patients, and reoperation was required in no patient for residual or recurrent spinal stenosis in the same segment(s) within 12–18 months. Adjacent level stenosis requiring decompression occurred in one Group 2 patient and two patients in Group 3. In five patients (three in Group 2 and two in Group 3) postoperative instability necessitated fusion surgery. Overall, there were no differences in the reoperation rate among groups.

### Patient Satisfaction

Patient satisfaction was higher in Group 1, with 7.5, 20, and 25% of patients displeased (in Groups 1, 2, and 3, respectively; *p* < 0.01). In the present randomized study, patient satisfaction was 96.4% during the 12- to 18-month follow-up period in Group 1 matched with 79.1 and 73.2% in Groups 2 and 3, respectively (*p* < 0.01) (Table 5).

**Table 5:** **Patient satisfaction following decompression of lumbar spinal stenosis**.

FU period	Group (%)		
	1	2	3
**3-Month**			
PSI (overall satisfaction w/op)	92.5	78.3*	77.2
satisfaction w/pain reduction	96.8	78.4*	71.3
satisfaction w/improved performance	90.1	72.3	72.5^‡^
**6-Month**			
PSI (overall satisfaction w/op)	91.3	77.2^†^	74.3^‡^
satisfaction w/pain reduction	95	81.3	79.4*
satisfaction w/improved performance	85.6	79.4*	78.3
**12-Month**			
PSI (overall satisfaction w/op)	96.4	79.1^†^	73.2^‡^
satisfaction w/pain reduction	98	78.5	72.1
satisfaction w/improved performance	88.3	68.3	65.4^‡^

*p < 0.05 compared with Group 1.

*^†^p < 0.01 compared with Group 1*.

*^‡^*p* < 0.001 compared with Group*.

## Discussion

Degenerative spinal stenosis is more often perceived in older people of age 60 and above (32–34). Laminectomy by bilateral partial facetectomy is the commonest way in the surgery of lumbar stenosis. While satisfactory in handling the symptoms of neurogenic claudication, the occurrence of new onset spondylolisthesis is reported to be as high as 31% (35). Radiographic evidence of the progression of spondylolisthesis was current if greater than 50% of the facet joint was resected at any one level (36).

A benefit of conventional laminectomy is that it offers good discernibility and adequate working space by removing posterior elements, including the spinous process, the supraspinous ligament and the interspinous ligament. The disadvantages of conventional laminectomy include the resection of osteoligamentous construction, which sometimes causes secondary spinal instability and trunk extensor weakness. The success percentage of the traditional laminectomy procedure is only 64%. This technique generates momentous intraoperative bleeding and has common surgical failures accredited to native tissue disturbance, incisional pain after surgery, sustained recovery time, and maybe failed back-surgery syndrome. The difficulties produced by iatrogenic spinal muscle damage are inevitable in patients experiencing operations to the lumbar spine (37, 38). Further minimally invasive methods might try to preserve osseous and ligamentous constructions, nevertheless these retained midline organizations, admittance to the nerve tissue and access to decompression in the lateral recesses. Similarly, it still needs undressing of the Para spinal musculature and the possible risk of neural injury in a small occupied space is also a problem, especially in patients with severe central stenosis (18, 37–39). Laminotomy has been described as positively treating characteristic stenosis (40). There are advantages and disadvantages of bilateral laminotomy above laminectomy. With laminotomy, the posterior ligamentous compound is secure and can remain to act as a tension band and additive to lumbar motion. But, a smaller resection of the posterior rudiments means there is a smaller operational space and may extend a case because of amplified practical difficulty. In addition, where there is a spinal fluid outlet, a complete laminectomy might be obligatory to see and repair the fee in the dura (5). In 1993, Postacchini et al. equaled bilateral laminotomy with laminectomy and determined that laminotomy is acceptable for mild-to-moderate stenosis, then laminectomy is favored when handling severe stenosis or spondylolisthesis. They recommend that with severe stenosis, bilateral laminotomy must not be routinely done. There are frequent issues that contribute to clinical policymaking. These are: severity of stenosis (41), segmental mobility before surgery (42), medicinal comorbidity (43), facet tropism (44), and liquid inside the facets (36).

Numerous authors have planned more personalized techniques, in particular bilateral and unilateral laminotomy for bilateral decompression, with a reported success rate of 60–80%. Bilateral and unilateral laminotomy prove advantageous for patients, with reduced postoperative pain, no additional fusion surgery and improved health-related quality-of-life (6, 39, 45–47). The trumpet laminectomy can diminish the muscle injury and has a benefit in that it conserves the inferior levels of the paraspinal muscle from atrophy. Shiraishi (48) and Watanabe et al. (38) described that splitting of the spinous process in laminectomy needs minimal separation of the muscles, which are protected from operative harm, and produces good outcomes. The trumpet laminectomy can be achieved for lumbar canal stenosis patients of all ages and at all levels of spinal canal stenosis without spondylolisthesis complication. The operation is generally a short surgical procedure and causes minimal intraoperative blood loss. The trumpet laminectomy micro decompression technique is still developing and needs more study to demonstrate its advantages and disadvantages in practice (28).

Thomé et al. reported a study in which 120 patients had undertaken lumbar canal stenosis decompression and were randomized to three treatment groups (bilateral laminotomy, unilateral laminotomy, and laminectomy). The total complication rate was lowest in patients who had experienced bilateral laminotomy. The least follow-up of 12 months occurred in 94% of patients.

Residual pain was lowest in Group 1 (VAS score 2.3 ± 2.4 and 4 ± 1 in Group 3; *p* = 0.05 and 3.6 ± 2.7 in Group 2; *p *=** 0.05). The Roland–Morris Scale score improved from 17 ± 4.3 before surgery to 8.1 ± 7, 8.5 ± 7.3, and 10.9 ± 7.5 (Groups 1–3, respectively; *p* = 0.001 compared with preoperative) corresponding to a dramatic increase in walking distance. In the majority of cases, bilateral laminotomy produced major advantages and thus established a possible treatment substitute (49).

In 2014, Henky et al. reported a study in which there were 62 patients with Canal stenosis. Out of the 62 patients, 62.9% had hypertrophy of the facet joint, 11.3% had granulation tissue, 79.1% had hypertrophy of the yellow ligament, and 64.5% had disc herniation. The typical procedure length was 68.9 min and intraoperative blood loss remained 47.4 ml. Intraoperative problems occurred in 3.2% of patients, through dural injury but without cerebrospinal fluid leakage. They reported that trumpet-type fenestration has a shorter duration, with minimal intraoperative blood loss (28).

In 2013, Yaman et al. reported the records of 40 patients who experienced surgical treatment for lumbar spinal stenosis by different methods, which were studied retrospectively. The patients were separated into two groups for the surgical procedure. In the first group, patients underwent classic laminectomy, while in the second group patients underwent bilateral decompression via a unilateral approach. Preoperative and postoperative computed tomography section areas of both groups were examined. VAS was used to evaluate low back and leg pain preoperatively and postoperatively at 1, 6, and 12 months. The two groups were compared in respect of surgery time and bleeding.

They concluded that bilateral decompression through a unilateral approach is an effective method without any instability effect, which provides sufficient decompression in the degenerative stenosis and increases patient comfort in the postoperative period (50).

Using these reports of effectiveness of these minimally invasive decompressions and based on our performance in these areas, we have offered the consequences of the first randomized prospective study to match the safety and conclusion of bilateral laminotomy compared with laminectomy and trumpet laminectomy in 120 patients with lumbar spinal stenosis. The frequency of complications did not vary meaningfully among the groups, while global perioperative morbidity was lowest afterward bilateral laminotomy. All three processes produced highly significant enhancement in symptoms and scores; but, a superior outcome was confirmed after bilateral laminotomy. Subsequently, the explanation of the bilateral laminotomy method (51), the authors of the clinical case series showed good results in 91% (29) at 1 year; 82% (12), 87% (52), 78% (53), and 68% (5) at 2 years; 85% (7) at 3 years, and 74% (54) at 6 years, in a prospective outcome study of 54 patients; Kleeman et al. (11) described good outcomes of 88% and patient satisfaction as 100% after 4 years without deteriorating (11).

In the present randomized study, patient satisfaction was 96.4% during the 12- to 18-month follow-up period in Group 1, which confirms the data of the above mentioned case series (49). These outcomes suggest that they are superior to laminectomy and trumpet laminectomy. Fusion was not indicated in Group 1 patients. If analysis of long-term follow-up data agrees with these results, bilateral laminotomy may prove more beneficial for patients with lumbar stenosis, lowering the need for additional fusion surgery.

Comparative preoperation and postoperation analysis designated significant escalation in computerized axial tomography section areas in three groups. However, the groups did not display any significant differences. Comparison of both area measurements showed no significant differences, which proves that sufficient decompression was safeguarded in the three approaches (50). Comparison of both area measurements and postoperation leg pain VAS scores indicate no significant differences, which reveals that sufficient decompression was ensured in bilateral decompression via the unilateral approach.

Although longer surgery time looks like a disadvantage compared to the classic procedure, surgery time for bilateral decompression has been perceived to decline as the surgeon improves his learning curve, as in our study. This is because of wide interest in the fenestration approach and, because we have a lot of experience in this, then our operation times for bilateral laminotomy are significantly low compared to two other groups and other studies (28, 49, 50). The practice of fine Kerrison rongeurs donated to the longer surgery time. To avoid complications, which might be caused by the high-speed burr, the surgeon had to act more industriously. Laminectomy is a simple decompressive procedure (6, 55), while bilateral laminotomy has been related to a prolonged operative duration (20, 24) and the unilateral approach has been thought technically more challenging (6). In our study, the duration of surgery was decreased in Group 1. There was no important difference between Groups 2 and 3. Khoo and Fessler (25) described an operative duration of 109 min for a single level micro endoscopic unilateral laminectomy and 88 min for an open laminectomy (25). Others have described smaller operative times for laminectomies (56), nonetheless records on laminectomy in which the facet joints are accurately secure are the outcome of less invasive surgery for lumbar stenosis.

Noticeably, the skin incision was longest in Group 3 and shortest in Group 1, which underlines the less invasive procedure of the laminotomy methods. Because all processes were done, relevant statistics in the literature are rare (57), while the importance of the cosmetic consequence has been harnessed (6). Blood loss was abridged in the bilateral laminotomy group (25, 57), but clinically unfavorable EBL necessary transfusions are actually infrequent in all decompressive techniques of lumbar stenosis (20, 57).

The authors of a clinical series linking bilateral laminotomy or trumpet laminectomy have found complication rates inferior or similar to laminectomy (5, 7, 8, 11, 17) but the sizes of the patient groups have been smaller and the studies were mostly retrospective or lacked a control group.

As a result, the main anxiety of spine surgeons, in view of less invasive techniques to decompress lumbar stenosis, has been an increased rate of neural injury (20, 22, 24). In the series reported by Verbiest (58), a postoperative increased radicular deficit was perceived in 5% of laminectomy-treated cases. Postacchini et al. (20) reported a postoperative increase in radiculopathy in one (1.3%) of 32 patients after laminectomy compared with three (11.5%) of 26 patients after bilateral laminotomy, but others have reported this complication in only 1% when using the latter approach (54). Referring to our data, definite injury to a nerve root did not happen. Intraoperative influence and/or compression of nerve roots, however, may aggravate radicular deficit.

In general, accidental durotomy rates for laminectomy have been revealed to range from 5 to 15% (59–61). Bilateral laminotomy is problematical by dural tears in 2 to 6% (5, 7, 11).

In our practice, three durotomies in the first and two durotomies in the second group were primarily repaired. When the complication rates were compared, the difference was not statistically significant. All dural tears were in the older patients, but the bilateral laminotomy approach does not bring extra risk to the elderly population (62).

The wound infection rate is about 2% of all spinal surgery cases (60, 63–65), and this complication was too infrequent in our study (2% in Groups 2 and 3).

For postoperative epidural hematomas, the occurrence ranges from 1 to 3% (7, 60). The incidence of postoperative hematomas after bilateral laminotomy must not vary from the low rates after micro discectomy, which agrees with our data.

In the current study, analysis of outcome was based on the VAS for pain, the ODI for disability and the PSI. Surgeon-based outcome measures were not considered. More importantly, however, the randomized study strategy minimized theoretical errors in the comparison of outcomes among groups. In our study, a minimum follow-up period of 12 months was obtainable for all patients. Symptoms and scores continued stable during that period. Yet, long-term follow up data are mandatory and will be sought.

Because we have less experience with surgical methods in trumpet surgery, this may be the reason for spending more time in the group who had trumpets surgery. However, the laminotomy surgery time was longer in other studies and we spent less time on this type of surgery. It seems that, in addition to selecting appropriate surgical technique for the treatment of any specific patient with spinal stenosis, knowledge of the surgeon and his experience will be useful in reducing surgical times and complications and therefore is more useful for recovery of clinical parameters. Perhaps our surgical team is very familiar with bilateral laminotomy, and this maybe the reason of some differences, such as the time of surgery or length of skin incision that had seen in this approach compared to other two surgical methods.

According to the results available, we recommended bilateral laminotomy in all patients with Spinal Stenosis regardless of age and severity of illness, as their preferred treatment.

## Conclusion

Bilateral laminotomy allows acceptable and safe decompression of the spinal canal in patients with lumbar stenosis. This was accompanied by a major benefit in most outcome factors during a minimum follow-up period of 1 year and is a current method with no instability effect, which offers sufficient decompression in the degenerative stenosis and increases patient comfort in the postoperative stage. Knowledge of the surgeon and his experience in surgical approaches will be useful in reducing surgical time and complications and therefore is more useful on recovery of clinical parameters.

## Ethics Statement

This study has been approved by the institutional review board of Mazandaran University of Medical Science, Sari, Iran, and written informed consent was obtained from the patients.

## Author Contributions

Designed study and wrote paper: KH; Collected data: HQ.

## Conflict of Interest Statement

The authors declare that the research was conducted in the absence of any commercial or monetary relationship that could be construed as a potential conflict of interest.

## Abbreviations

LSS, lumbar spinal stenosis; ODI, oswestry disability index; VAS, visual analog scale.
